# Progesterone promotes CXCl2-dependent vaginal neutrophil killing by activating cervical resident macrophage–neutrophil crosstalk

**DOI:** 10.1172/jci.insight.177899

**Published:** 2024-10-22

**Authors:** Carla Gómez-Oro, Maria C. Latorre, Patricia Arribas-Poza, Alexandra Ibáñez-Escribano, Katia R. Baca-Cornejo, Jorge Gallego-Valle, Natalia López-Escobar, Mabel Mondéjar-Palencia, Marjorie Pion, Luis A. López-Fernández, Enrique Mercader, Federico Pérez-Milán, Miguel Relloso

**Affiliations:** 1 Laboratorio de InmunoReproducción, Grupo Fisiopatología de la mujer, del embarazo, parto y puerperio, Instituto de Investigación Sanitaria Gregorio Marañón (IiSGM), Madrid, Spain.; 2 Departamento de Microbiología y Parasitología, Facultad de Farmacia, Universidad Complutense de Madrid, Madrid, Spain.; 3 Laboratorio de InmunoRegulación, IiSGM, Madrid, Spain.; 4 Laboratorio de Farmacogenética, Grupo de Farmacia Hospitalaria y Farmacogenómica, IiSGM, Madrid, Spain.; 5 Unidad Cirugía Endocrino-metabólica, Servicio de Cirugía General y Aparato Digestivo, Hospital General Universitario Gregorio Marañón, Madrid, Spain.; 6 Unidad de Reproducción Asistida, Servicio de Obstetricia y Ginecología, Hospital General Universitario Gregorio Marañón, Madrid, Spain.

**Keywords:** Endocrinology, Infectious disease, Bacterial infections, Innate immunity, Macrophages

## Abstract

Vaginal infections in women of reproductive age represent a clinical dilemma with significant socioeconomic implications. The current understanding of mucosal immunity failure during early pathogenic invasions that allows the pathogen to grow and thrive is far from complete. Neutrophils infiltrate most tissues following circadian patterns as part of normal repair, regulation of microbiota, or immune surveillance and become more numerous after infection. Neutrophils are responsible for maintaining vaginal immunity. Specific to the vagina, neutrophils continuously infiltrate at high levels, although during ovulation, they retreat to avoid sperm damage and permit reproduction. Here we show that, after ovulation, progesterone promotes resident vaginal macrophage–neutrophil crosstalk by upregulating Yolk sac early fetal organs (FOLR2^+^) macrophage CXCl2 expression, in a TNFA-patrolling monocyte-derived macrophage–mediated (CX3CR1^hi^MHCII^hi^-mediated) manner, to activate the neutrophils’ capacity to eliminate sex-transmitted and opportunistic microorganisms. Indeed, progesterone plays an essential role in conciliating the balance between the commensal microbiota, sperm, and the threat of pathogens because progesterone not only promotes a flurry of neutrophils but also increases neutrophilic fury to restore immunity after ovulation to thwart pathogenic invasion after intercourse. Therefore, modest progesterone dysregulations could lead to a suboptimal neutrophilic response, resulting in insufficient mucosal defense and recurrent unresolved infections.

## Abstract 2

We characterized mouse cervical resident macrophage subsets in different hormonal contexts and in the presence of a variety of pathogens. Progesterone up-regulates cervical macrophage crosstalk to promote neutrophil-killing competency.

## Introduction

Vaginal infections have become a major worldwide public health concern in women of reproductive age, posing not only an epidemiological challenge but also contributing to infertility, preterm birth, miscarriages, and the increase of other infectious diseases (1). Although some have not yet been determined, the main causative agents of symptomatic vaginal infections are 20%–25% candida, 40%–50% bacterial vaginosis, and 15%–20% trichomonas (2, 3). Mixed vaginal infections affect more than 20% of women, while recurrent vulvovaginal candidiasis affects approximately 8% of women worldwide (1, 4). Despite the efforts of the World Health Organization (WHO), cases of sexually transmitted and recurrent vaginal infections are persisting and rising. The WHO has reported an estimated 376 million new cases of chlamydia, gonorrhoea, syphilis, and trichomoniasis. Notably, there was an almost 30% rise in the incidence of chlamydia, gonorrhoea, and syphilis between 2015 and 2019 among all cases of vaginal infections, significantly contributing to the overall burden that these present (3). Unfortunately, obstacles such as the lack of public awareness, misdiagnosis, erroneous antimicrobial treatment, resistance, or limited knowledge regarding pathogen-mucus interactions continue to impede efforts to control these pathogens (5). Over the past decades, studies have mainly focused on preestablished vaginal infections; however, the current understanding of how mucosal immunity failure allows pathogens to colonize during the early stages remains far from clear.

Female mucosa must maintain the reproductive milieu during ovulation, as both pathogens and host neutrophils affect sperm quality. Sex hormones intricately regulate the cervical role in female reproduction by blocking the ascent of pathogens while allowing sperm passage. Furthermore, the cervical mucosa serves as the main point of entry for neutrophils into the vaginal lumen, where they are the predominant line of cellular defense (6–8) and are highly efficient at killing pathogens (9, 10). Specific to cervical tissue, neutrophil migration is exceptionally high, continuous, and dependent on the ovarian cycle stage, but it is independent of microbial invaders or sperm (8). During ovulation, estradiol (E2) reduces neutrophil extravasation to favor reproduction over immune defense because sperm are fragile cells and small amounts of neutrophils could be detrimental to them (11, 12). However, progesterone (P4) reestablishes neutrophil influx to the vaginal lumen after ovulation (8, 12). The activation of neutrophils into microbicidal cells must be tightly regulated, since their uncontrolled activity can be damaging to the surrounding tissue (13), even killing sperm (12).

Heterogeneous pools of tissue-resident macrophages are one of the most important inflammatory regulators (14–19) that attract neutrophils to the focal point of inflammation (reviewed in refs. 20, 21). Macrophages through cross-communication systems (22) regulate the high destructive potential of Neutrophils. In order to perform such diverse roles, macrophages are composed of different populations with distinct origins, each possessing diverse functions in response to the tissue microenvironment or upon encountering inflammatory cues (23, 24). In most tissues, the presence of microbes induces inflammation that results in cell stress and death of resident macrophages. Macrophages maintain their own homeostasis within the tissue, compensating for reductions in one population by increasing another (25). This dynamic balance allows for the clearance of damaged cells and, in the case of infection, promotes the elimination of the responsible pathogen (26). Not all macrophage populations replenish in the same way. Yolk sac–derived macrophages (YsM/FOLR2; F480^+^FOLR2^++^CCR2^–^MHCII^+^CX3CR1^+^) exhibit robust proliferation and selfrenewal within the tissue (27). In contrast, monocyte-derived macrophages replenish from circulating blood monocytes (28). In mice, 2 distinct monocyte populations infiltrate tissues under different conditions, giving rise to 2 macrophage subsets. Inflammatory monocytes (Mo-inf) recruited via the CCR2/CCL2 axis predominantly contribute to inflamed tissues, forming the inflammatory monocyte-derived macrophage (MdM-Inf/CCR2; F480^+^FOLR2^–^CCR2^++^MHCII^++^CX3CR1^++^) population (25, 29). Conversely, patrolling monocytes (Mo-pat) selectively migrate to noninflamed sites through the CX3CR1/Cx3cl1 axis, establishing residence as patrolling monocyte-derived macrophages (MdM-pat/MHCII^hi^ or CX3CR1^hi^) (F480^+^FOLR2^–^CCR2^–^MHCII^+++^CX3CR1^+++^) for surveillance functions (28, 30) (for markers, review Supplemental Figure 1A; supplemental material available online with this article; https://doi.org/10.1172/jci.insight.177899DS1) (23, 24, 31–34).

Cervical immune responses must facilitate positive reproductive function while protecting against sexually transmitted pathogens or unresolved opportunistic infections. Cervical macrophage numbers are relatively stable throughout the menstrual cycle, with a slight increase in the cervical mucosa during the menstrual phase (for review, see refs. 35, 36). However, their functional specialization in reproductive events, where they are expected to function effectively as innate immune effectors without interfering with reproduction, remains undefined. In the present manuscript, we describe the characterization of tissue-resident macrophages in the cervix and report their interactions with neutrophils under distinct hormonal contexts, challenged with a variety of pathogens (*Neisseria gonorrhoeae*, *Trichomonas vaginalis*, and *Candida albicans*) to allow for both reproduction and protection from invading pathogens.

## Results

### Ovarian cycle effect on the neutrophil-killing capacity in the vagina.

**Figure 1:**
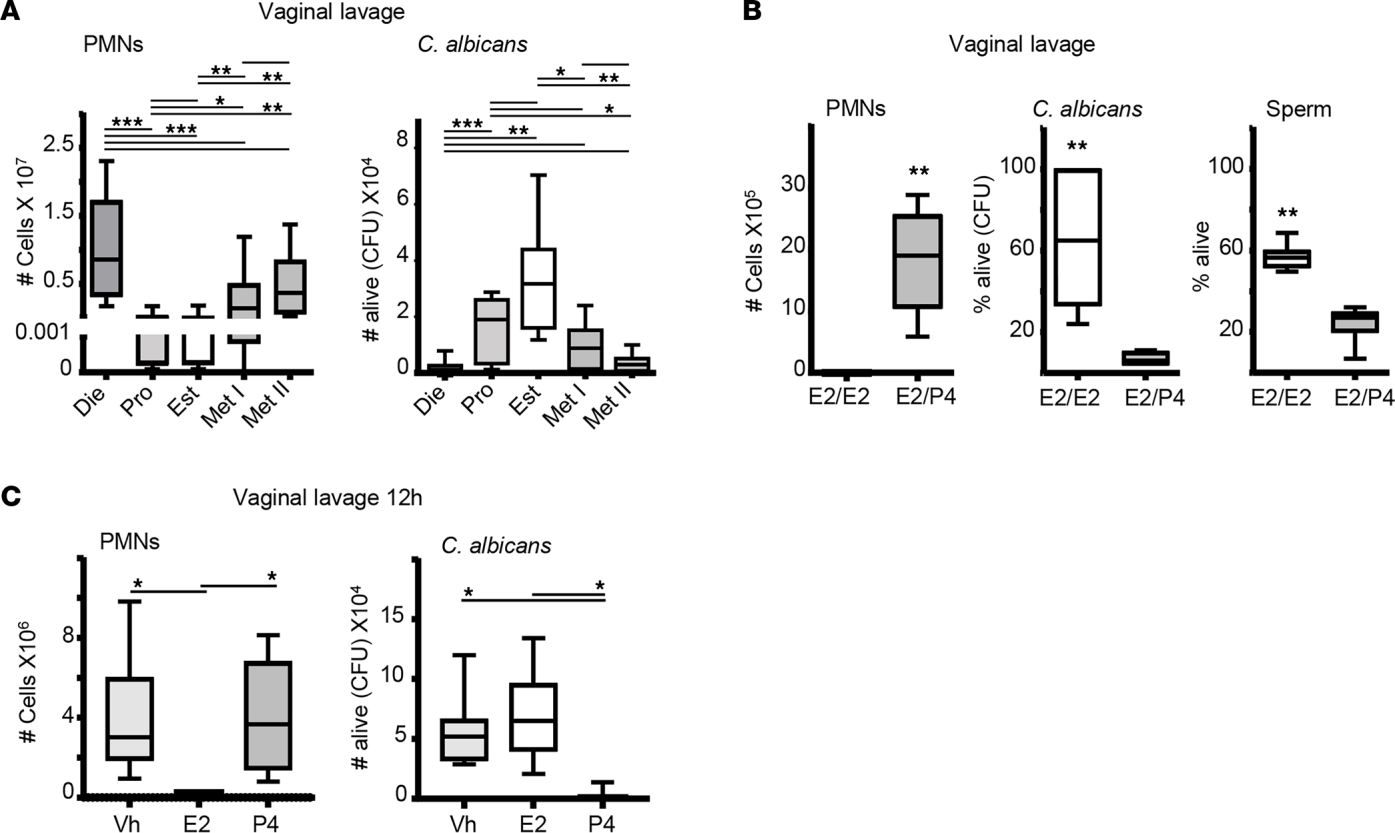
Neutrophil killing in the vaginal lumen. (**A**) Adult female mice selected by vaginal smear and challenged in the vagina with *C. albicans*. Number of neutrophil and fungal burden (CFU) in the vaginal lavage. Kruskal-Wallis analysis (*n* = 8–12 mice per group). (**B**) Ovariectomized mice treated with estradiol or progesterone to mimic the ovarian cycle. Number of neutrophils and percentage of live *C*. *albicans* (CFU) in the vaginal lavage 12 hours after challenge (*n* = 7–8 mice per group, Mann-Whitney *U* test). Percentage of live sperm in the vaginal PMNs/sperm coculture (*n* = 3 experiments, Mann-Whitney *U* test). (**C**) Estradiol-, progesterone-, or vehicle-treated mice challenged in the vagina with *C*. *albicans* for 12 hours. Number of neutrophils analyzed by flow cytometry and *C. albicans* by CFU. Data were calculated in at least 3 experiments (*n* = 7–9 mice per group, Mann-Whitney *U* test) and expressed as box and whiskers at 10–90 percentiles. *P < 0.05, **P < 0.01, and ***P < 0.001. Die, diestrus; Pro, proestrus; Est, estrous; Me I, Metestrus I; and Me II, Metestrus II; PMN, polymorphonuclear leukocytes; CFU, colony-forming unit; Vh, vehicle; E2, estradiol; P4, progesterone.

Neutrophils are the most abundant leukocyte in the vaginal lumen, and their numbers fluctuate depending on the stage of the ovarian cycle (8, 37, 38). During proestrus and estrus (E2 follicular and ovulatory phase), neutrophil numbers decrease in the vaginal lumen and increase during metestrus and diestrus (P4 luteal phase). To test the ovarian cycle’s effect on neutrophil-killing efficiency, we challenged mice with equal amounts of *C*. *albicans* at different stages of the ovarian cycle. We detected higher *C*. *albicans* numbers in estrus (multiplicity of infection [MOI] 50) and proestrus (MOI 20) than in metestrus (MOI 0.2) and diestrus (MOI 0.1), which seems consistent with the pathogen/neutrophil (MOI) ratio prior to infection (Figure 1A). To study how sex hormones balance immunity and reproduction, we mimicked estrus by treating ovariectomized mice with E2 and administered a sequential treatment of E2 and P4 to mimic metestrus (10). Thereafter, we challenged them with *C*. *albicans* or sperm. We detected lower neutrophil numbers (MOI 200) and more live *C*. *albicans* or sperm (~10-fold and 3-fold) in E2/E2-treated mice (estrus) than in E2/P4-treated ones (MOI 1) (Figure 1B), suggesting that P4 could upregulate neutrophil numbers and their killing capacity as well as confirming that this experimental procedure mimics the physiological state.

To investigate the role of E2 and P4, we treated ovariectomized mice with vehicle (Vh), E2, or P4 and challenged them with *C*. *albicans*. Consistently, E2-treated mice (MOI 12) failed to control the fungal infection, while P4-treated mice (MOI 0.6) eliminated it; surprisingly, Vh-treated mice did not prevent infection despite having the same ratio (MOI 0.6) as P4-treated mice (Figure 1C), suggesting that P4 enhances the killing ability of neutrophils. We hypothesized that these hormones might affect the neutrophil-killing capacity. Therefore, we proceeded to treat ex vivo neutrophils with Vh, E2, and P4 and infected them with *C*. *albicans* (MOI 1). We observed that neutrophil killing was unaffected by direct hormonal treatment (Supplemental Figure 1B) and thus concluded that sex hormones regulated neutrophil-killing capacity in vivo by other indirect mechanisms (36).

### MdM-pat depletion inhibits P4-treated neutrophil killing but does not affect neutrophil extravasation.

**Figure 2:**
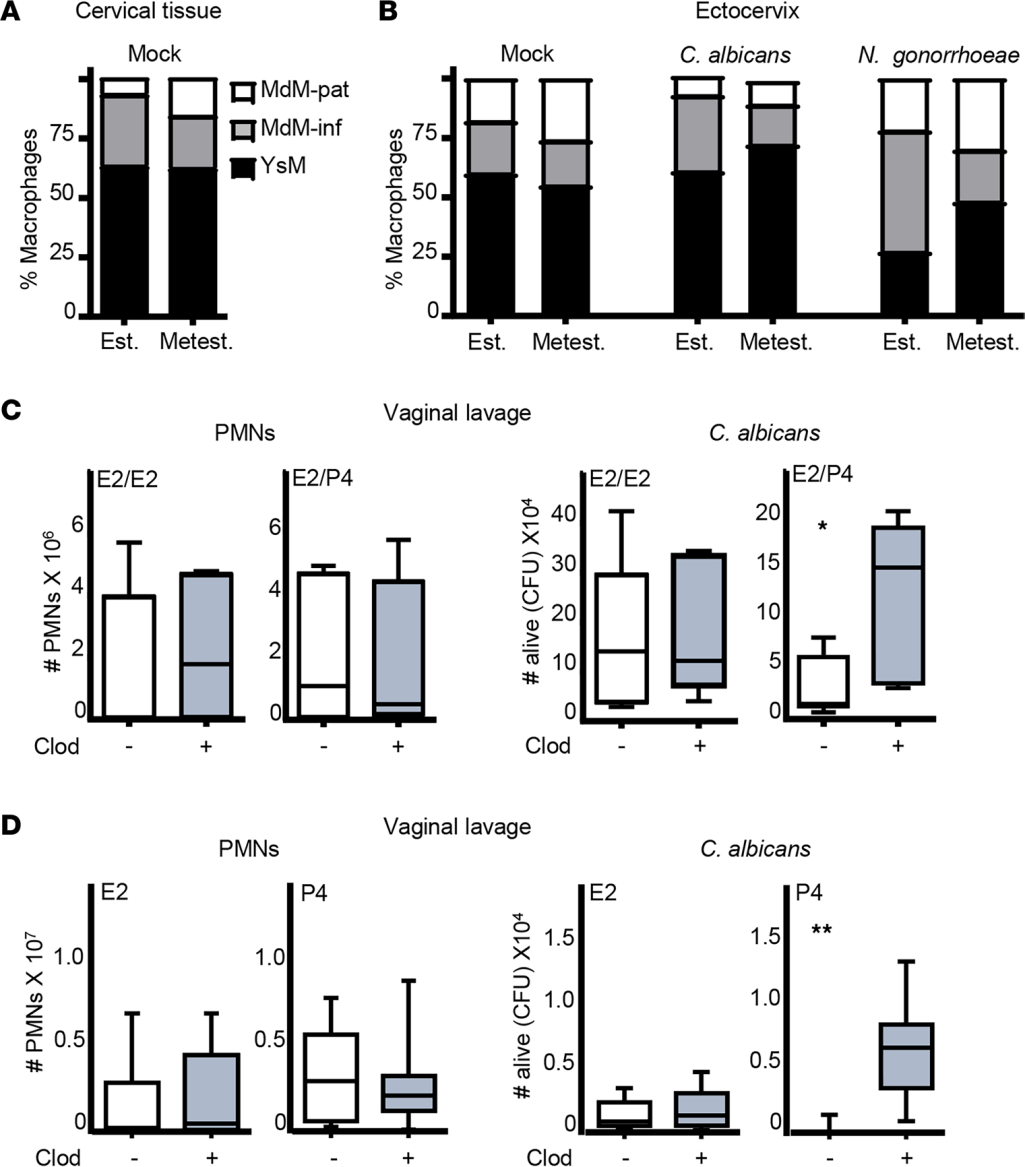
Macrophage subset density and neutrophil killing in the monocytes/MdM-pat depletion model. (**A** and **B**) Stacked bar chart representation of macrophage subset density (data from Supplemental Figures 2, C and D) in the vaginal tissue by flow cytometry (**A**) and cervix by confocal microscopy (**B**). (**C**) Ovariectomized mice were administered clodronate liposomes along with estradiol, either in combination with additional estradiol or progesterone, to simulate the ovarian cycle. Subsequently, mice were subjected to a vaginal challenge with *C. albicans*. The E2/P4- and E2/E2-treated mice were analyzed at 12 and 24 hours after challenge, respectively. Mann-Whitney *U* test (*n* = 5–9 mice per group). (**D**) Mice treated with clodronate liposomes, single hormone and challenged in the vagina with *C*. *albicans*. Mice treated with P4 and E2 were assessed at 12 and 24 hours after the challenge, respectively. Number of neutrophils analyzed by flow cytometry and live *C*. *albicans* (CFU) in the vaginal lavage. Confocal data were calculated in 2–4 different sections of each sample. Mann-Whitney *U* test (*n* = 7–14 mice per group). Data were calculated from at least 3 experiments and expressed as box and whiskers, at 10–90 percentiles. *P < 0.05 and **P < 0.01. YsM, Yolk sac–derived macrophages (F4/80-FOLR2); MdM-inf, inflammatory monocyte–derived macrophages (F4/80-CCR2); MdM-pat, patrolling monocyte–derived macrophages (F4/80-MHCII/CX3CR1); PMNs, polymorphonuclear leukocytes; CFU, colony-forming unit; Clod, clodronate; E2, estradiol; P4, progesterone; Est, estrous; and Metest, metestrus.

We hypothesized that P4 could activate macrophage-neutrophil crosstalk to promote neutrophil-killing capacity because macrophages activate neutrophils by cooperative induction of cytokine (TNFA, CXCL1, CXCl2, etc.) expression (22). First, we confirmed that cervical macrophages express E2 (Esr1) and P4 receptors (PGR) (Supplemental Figure 1C) (35, 36). Then, we detected the same density of cervical macrophages (~55% of CD45^+^ cells) in estrus and metestrus mock or infected mice (Supplemental Figure 1D and Supplemental Figure 2, A and B). Subsequently, we proceeded to analyze the most common subsets of macrophages, delineated by their respective origins (23, 24, 31–34). Our findings reveal that, in the cervical tissue, MdM-pat were mostly positioned underneath the epithelial layer (35) while YsM and MdM-inf were located in the submucosal vascular plexus. YsM were the most common cervical macrophages (~60% of F4/80 cells). Moreover, we found higher numbers of MdM-pat in metestrus and MdM-inf in estrus; however, Mo-inf (LY6C^+++^CCR2^++^CXCR1^–^CX3CR1^+^) and Mo-pat (LY6C^++^CCR2^+^CXCR1^+^CX3CR1^++^) were unaffected by the ovarian cycle stage. Next, mice in estrus and metestrus were challenged with *C*. *albicans* or *N*. *gonorrhoeae* and metestrus cervical tissues exhibited a more significant (*P* = 0.002 and 0.001) presence of YsM (Figure 2, A and B; Supplemental Figure 2, C and D; and Supplemental Figure 3A). Hence, the presence of MdM-pat within cervical tissue is higher during metestrus and appears to be unaffected by inflammatory stimuli. Notably, we observed a heightened density of YsM during infections.

To assess the role of MdM-pat in neutrophil killing, we depleted monocytes via clodronate liposome treatment. Clodronate liposomes administered intravascularly transiently deplete blood and perivascular populations of monocytes/macrophages. Mo-inf rapidly return to normal levels, whereas Mo-pat have delayed recovery kinetics (34, 39, 40) (Supplemental Figure 3B). After this, mice were treated with E2/E2 and E2/P4 to mimic the ovarian cycle and were challenged with *C*. *albicans*. There were no significant alterations observed in the overall macrophage populations. However, we detected a significantly reduced (~2-fold) MdM-pat density, whereas E2/P4-treated mice increased the YsM population (Supplemental Figure 3, C–F). We observed the same number of vaginal neutrophils in both mock and clodronate-treated mice in both ovarian cycle stages, which allowed us to analyze the role of MdM-pat on neutrophil killing. Clodronate treatment drastically reduced (~7-fold) the neutrophils’ candidacidal capacity in E2/P4-treated mice. Interestingly, E2/E2-treated mice were unable to kill candida either way (Figure 2C). Therefore, we hypothesized that MdM-pat and P4 lead to the increase in neutrophil killing. Mice were then treated with either E2 or P4, and we observed that clodronate treatment had no effect on vaginal neutrophil migration but consistently reduced (~6-fold) neutrophil killing in P4-treated mice (Figure 2D). Therefore, we concluded that vaginal neutrophils from E2-treated mice were unable to kill candida even when neutrophils were present in the vaginal lumen. However, neutrophils from P4-treated mice were highly efficient at killing candida in a P4- and MdM-pat–dependent manner. Therefore, we focused on the effects of P4 on MdM-pat.

### Hormonal regulation of cervical MdM-pat TNFA expression.

**Figure 3:**
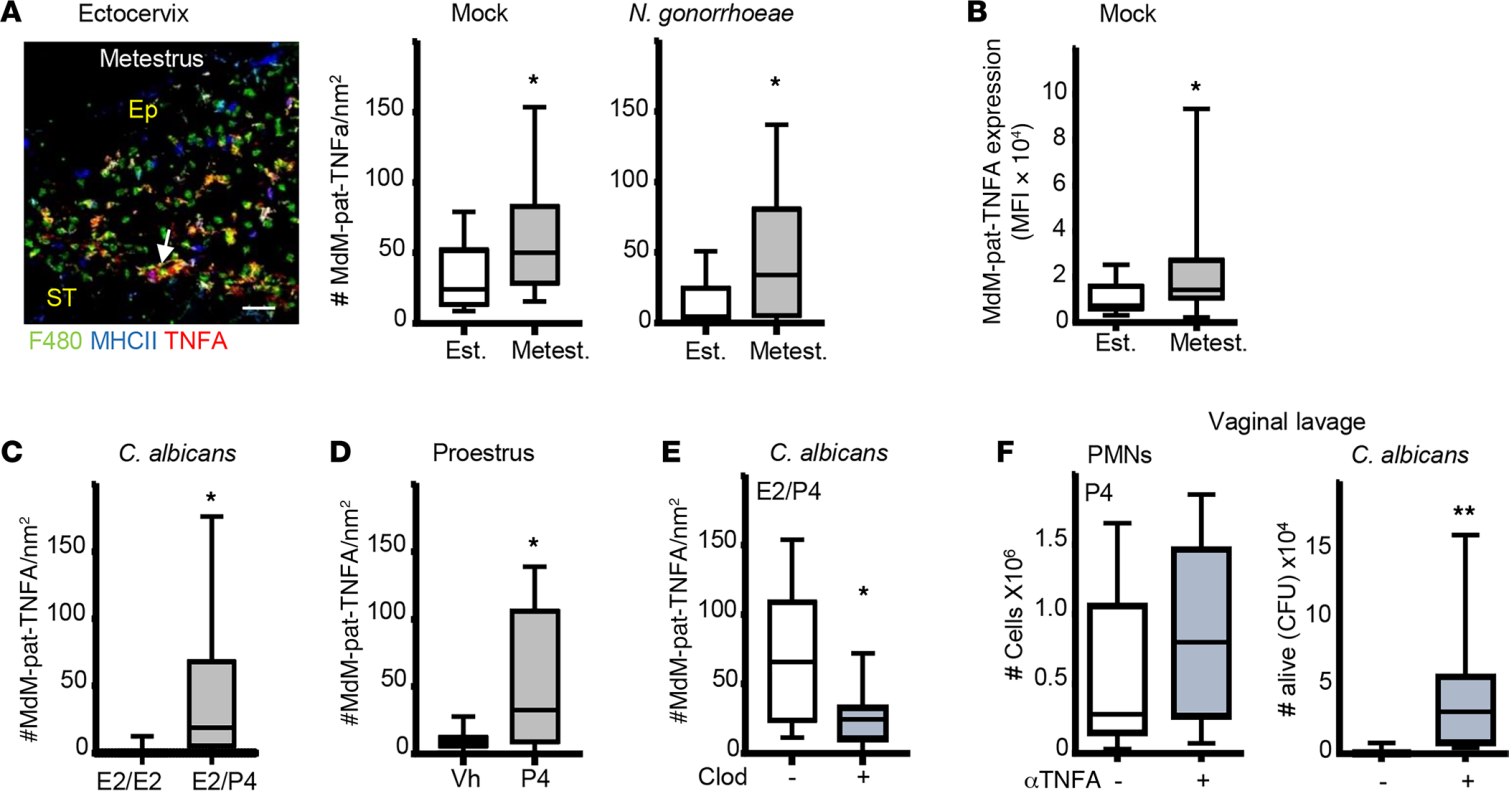
TNFA expression in MdM-pat in the ectocervix during the ovarian cycle. (**A**) Photomicrograph of the ectocervix of metestrus mice selected by vaginal smear. White arrow points to TNFA expression on MdM-pat (F4/80-MHCII). Quantification of TNFA expression in cervical MdM-pat (F4/80-CX3CR1-MHCII) mock- and *N*. *gonorrhoeae*–infected by confocal microscopy. Mann-Whitney *U* test (*n* = 6–9 mice per group). (**B**) TNFA expression in MdM-pat (F4/80-MHCII) in the vaginal tissue assayed by flow cytometry. Mann-Whitney *U* test (*n* = 10 mice per group). (**C**) TNFA expression in cervical MdM-pat (F4/80-CX3CR1-MHCII) sections of ovariectomized estradiol-treated mice treated with estradiol or progesterone to mimic the ovarian cycle. Mann-Whitney *U* test (*n* = 6 mice per group). (**D**) TNFA expression in cervical MdM-pat (F4/80-CX3CR1-MHCII) from proestrus mice (selected by vaginal smear) treated with progesterone. Mann-Whitney *U* test (*n* = 6 mice per group). (**E**) TNFA expression in cervical MdM-pat (F4/80-CX3CR1-MHCII) from progesterone-treated ovariectomized mice and injected with clodronate liposomes. Mann-Whitney *U* test (*n* = 8 mice per group). (**F**) Ovariectomized mice treated with progesterone and TNFA inhibitor (etanercept, 3 mg/kg) challenged in the vagina with *C*. *albicans*. Number of neutrophils analyzed by flow cytometry and number of live *C*. *albicans* (CFU) in the vaginal lavage 12 hours after challenge. Confocal data were calculated in 2 –4 different sections of each sample. Mann-Whitney *U* test (*n* = 12 mice per group). Data expressed as box and whiskers, at 10–90 percentiles. *P < 0.05. Scale bar: 50 μm. Vh, vehicle; E2, estradiol; P4, progesterone; MdM-pat, patrolling monocyte–derived macrophages; Clod, clodronate; Ep, epithelium, St, Stroma; Est, estrous; and Metest, metestrus.

Given that Mo-pat depletion resulted in the abolition of neutrophil-killing activity, our focus shifted toward examining MdM-pat cytokine production in the cervix. We assayed for several MdM-pat cytokine expressions (41) and detected a significant increase of MdM-pat TNFA expression in metestrus compared with estrus mice by confocal microscopy in *N*. *gonorrhoeae–*infected and noninfected mice (Figure 3A and Supplemental Figure 4A) and by flow cytometry (~3-fold; Figure 3B), whereas YsM and MdM-inf did not show this effect (Supplemental Figure 4B). Therefore, we hypothesized that P4 could upregulate MdM-pat TNFA expression in the cervix. To test that, we treated ovariectomized mice with E2 and P4 and detected higher (~3-fold) TNFA expression in E2/P4- versus E2/E2-treated mice that were *C*. *albicans* challenged (Figure 3C). Next, we treated proestrus-stage mice (high E2 and low P4) with P4 (Prolutex) and found a significant increase (~5-fold) of TNFA expression in MdM-pat noninfected mice (Figure 3D). Hence, we concluded that P4 is the key factor responsible for the upregulation of MdM-pat TNFA expression. To demonstrate the effect of MdM-pat on TNFA expression, we depleted Mo-pat cells using clodronate treatment (Supplemental Figure 3B). As a result, we observed a significant decrease in MdM-pat cells expressing TNFA (Figure 3E) in E2/P4-treated mice. This reduction is likely attributed to the previously reported decrease in MdM-pat cell numbers caused by clodronate treatment (Supplemental Figure 3D). Moreover, we treated P4-mice with etanercept (TNFA inhibitor) and detected a drastic inhibition (~600-fold) of neutrophil-killing capacity compared with nontreated mice, although vaginal neutrophil numbers remained the same (Figure 3F). To validate TNFA’s role in neutrophil killing regulation, we infected ex vivo neutrophils with candida and treated them with different concentrations of TNFA. Surprisingly neutrophil candidacidal activity remained unaffected (Supplemental Figure 4C). Thus, we hypothesized that TNFA could indirectly regulate the expression of other factors that mediate the killing capabilities of neutrophils.

### P4 regulation of the MdM-pat and YsM crosstalk to activate neutrophil-killing capacity.

**Figure 4:**
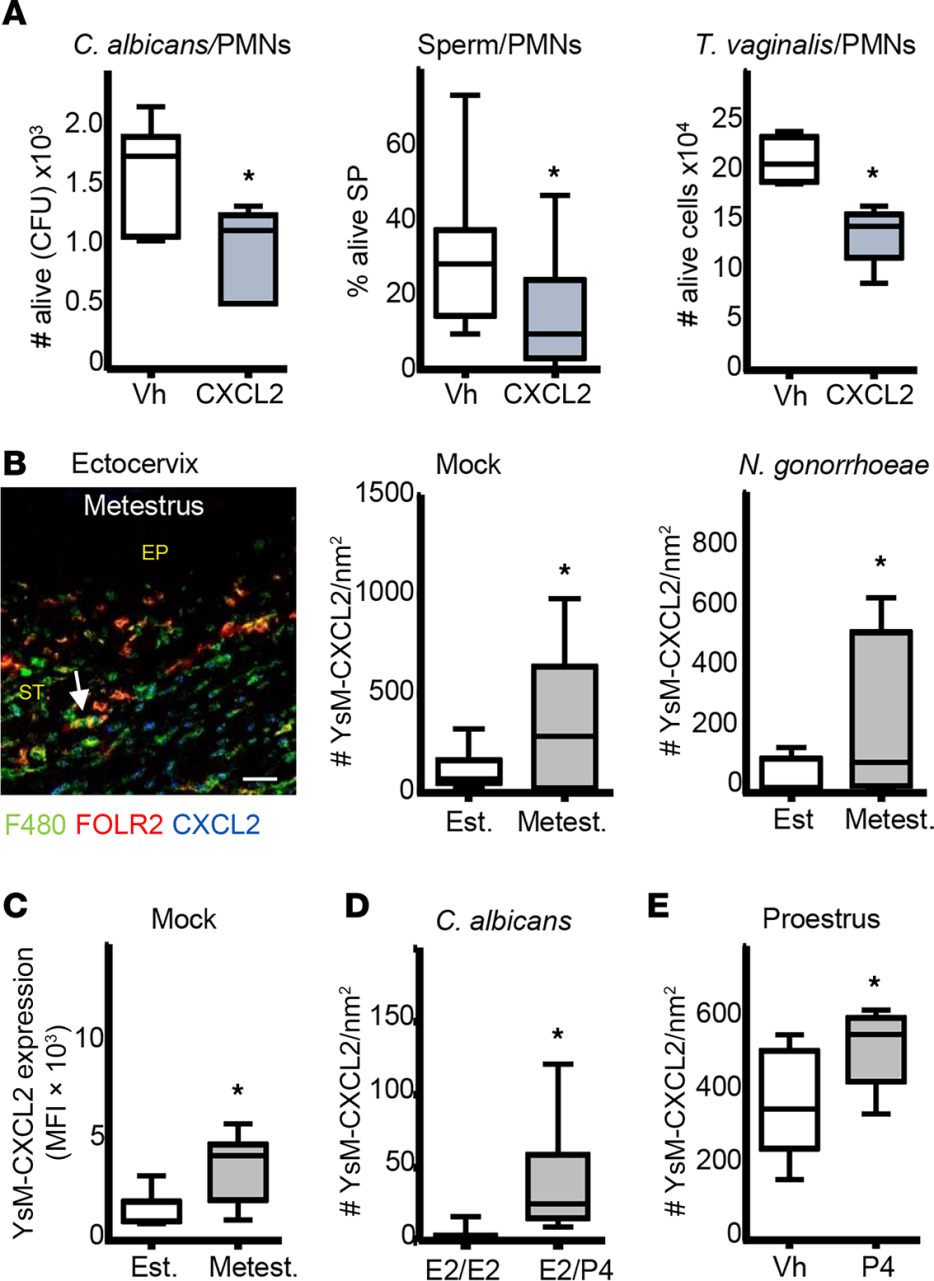
CXCl2 expression in YsM in the ectocervix during the ovarian cycle. (**A**) Live *C. albicans,* sperm, or *T. vaginalis* coculture with ex vivo neutrophils treated with CXCl2 (30 ng/mL). Representative experiment of 3 technical repeats. Mann-Whitney *U* test. (**B** and **C**) Photomicrograph of the ectocervix of metestrus mice selected by vaginal smear. White arrow points to CXCl2 expression on YsM (F4/80-FOLR2). Quantification of CXCl2 expression in YsM (F4/80-FOLR2) mock- and *N*. *gonorrhoeae*–infected by confocal microscopy and flow cytometry. Mann-Whitney *U* test (*n* = 6–9 mice per group). (**D**) CXCl2 expression in YsM (F4/80-FOLR2) from cervical sections of ovariectomized estradiol-treated mice treated with estradiol or progesterone to mimic the ovarian cycle. Mann-Whitney *U* test (*n* = 4 mice per group). (**E**) CXCl2 expression in cervical YsM (F4/80-FOLR2) from proestrus mice treated with progesterone (*n* = 5–6 mice per group). Confocal data were calculated in 2–4 different sections of each sample. Mann-Whitney *U* test. Data expressed as box and whiskers, at 10–90 percentiles. *P < 0.05 Mann-Whitney *U* test. Scale bar: 50 μm. Vh, vehicle; E2, estradiol; P4, progesterone; YsM, Yolk sac–derived macrophages; Ep, epithelium; St, stroma; Clod, clodronate; Est, estrous; Metest, metestrus; CFU, colony-forming unit.

We hypothesized a coordinated TNFA/CXCl2 axis interplay between macrophages (42) in the context of P4 to regulate neutrophils. MdM-pat could release TNFA to upregulate CXCl2 expression in macrophages; this might be necessary for neutrophils to resolve the infection. First, we tested whether CXCl2 could modify neutrophil-killing capacity by treating ex vivo neutrophils with CXCl2 and infecting them with *C*. *albicans*, sperm, or *T*. *vaginalis*. We detected a significant increase (~3-fold) in their killing activity capacity and found that CXCl2 did not affect pathogens viability (Figure 4A and Supplemental Figure 4D). Secondly, we analyzed CXCl2 expression in the 3 macrophage subpopulations and detected an increase of YsM CXCl2 expression in metestrus compared with estrus mice by confocal microscopy (~8-fold) and flow cytometry (~4-fold) in *N*. *gonorrhoeae*–infected and uninfected mice (Figure 4, B and C). In contrast, CXCl2 expression in Mdm-pat and MdM-inf macrophages remained unchanged (Supplemental Figure 4E). Next, to investigate whether P4 could enhance YsM CXCl2 expression in the cervix, we administered E2 and P4 to ovariectomized mice. Our findings reveal significantly higher CXCl2 expression (~5-fold) in YsM E2/P4-treated mice when compared with E2/E2-treated mice infected with *C. albicans*. Additionally, proestrus mice treated with P4 exhibited an increase (~2-fold) in YsM CXCl2 expression even in the absence of infection (Figure 4, D and E). Thus, we hypothesize that P4 could upregulate CXCl2 expression in YsM by stimulating MdM-pat to express TNFA.

**Figure 5:**
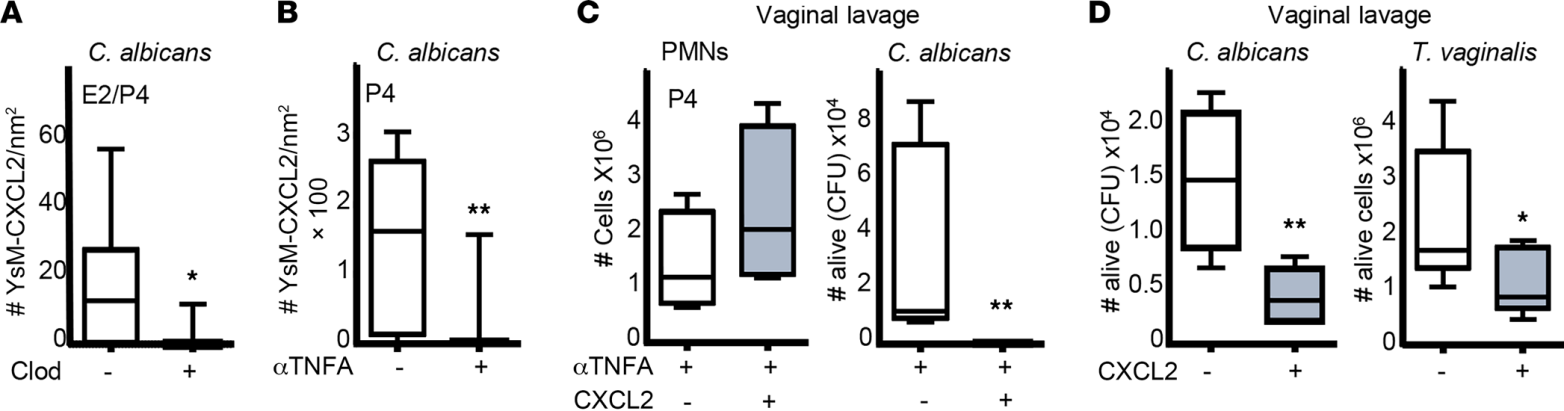
P4 upregulates neutrophil killing competency by inducing TNFA expression in MdM-pat, which induces YsM to produce CXCl2. (**A**) CXCl2 expression in cervical YsM (F4/80-FOLR2) from ovariectomized estradiol- and progesterone-treated mice injected with clodronate liposomes 12 hours after the *C. albicans* challenge. Mann-Whitney *U* test (*n* = 5–6 mice per group). (**B**) CXCl2 expression in YsM (F4/80-FOLR2) in ovariectomized mice treated with progesterone and TNFA inhibitor (etanercept, 3 mg/kg) challenged in the vagina with *C*. *albicans*, analyzed via confocal microscopy using cervical sections. Mann-Whitney *U* test (*n* = 8 mice per group). (**C**) Progesterone and TNFA inhibitor–treated mice challenged in the vagina with *C*. *albicans* and treated with CXCl2 (160 ng) in the vagina 2 hours after the infection. Mann-Whitney *U* test (*n* = 7 mice per group). (**D**) Ovariectomized mice challenged in the vagina with *C*. *albicans* or *T*. *vaginalis* and treated with CXCl2 (160 ng) 2 hours after the infection. Number of live *T*. *vaginalis* cells by trypan blue staining and *C*. *albicans* fungal burden (CFU) in the vaginal lavage 12 hours after the challenge. Mann-Whitney *U* test (*n* = 8–10 mice per group). Confocal data were calculated in 2–4 different sections of each sample. Data were calculated in at least 3 experiments and expressed as box and whiskers, at 10–90 percentiles. *P < 0.05 and **P < 0.01. Mann-Whitney *U* test. Scale bar: 50 μm. CFU, colony-forming unit; YsM, Yolk sac–derived macrophages.

To demonstrate that cervical MdM-pat TNFA expression could regulate YsM’s secretion of CXCl2, we analyzed CXCl2 expression in clodronate-treated mice and detected a significant reduction (~5-fold) in YsM CXCl2 expression in the E2/P4-treated mice (Figure 5A). In addition, we treated P4 mice with etanercept (TNFA inhibitor) and detected lower YsM CXCl2 expression (~100-fold) in the cervix (Figure 5B), consistent with the drastic inhibition (~600-fold) of neutrophil-killing capacity in those mice (Figure 3F). In summary, our research findings suggest that P4 upregulates MdM-pat TNFA expression. Consequently, this TNFA induction in MdM-pat leads to the secretion of CXCl2 by YsM, ultimately promoting the activation of the neutrophil-killing program. To test this hypothesis, we treated mice with etanercept and added CXCl2 in the vagina 2 hours after the *C*. *albicans* challenge and detected a lower number (~85-fold) of live *C*. *albicans* and nonsignificant differences of neutrophils quantity (Figure 5C). Therefore, we conclude that P4 induces TNFA expression in MdM-pat, which in turn induces YsM to produce CXCl2 to activate cervical neutrophil-killing competency. Thus, P4 plays a central role in upregulating cervical-macrophage-neutrophil crosstalk to favor immunity after ovulation.

### CXCl2 enhances neutrophil-killing capability.

Our data also suggest that CXCl2 is a key regulator of neutrophil-killing capability. Hence, we questioned whether CXCl2 treatment alone could activate the in vivo microbicidal capacity of neutrophils. We, therefore, ovariectomized mice to remove the source of sex hormones, challenged them with *C*. *albicans* or *T*. *vaginalis,* and treated them intravaginally with CXCl2 two hours after the challenge. We detected a significant increase (~3-fold) in neutrophil-killing capacity — although neutrophil numbers did not change and CXCl2 did not affect pathogen viability (Figure 5D, Supplemental Figure 4D, and Supplemental Figure 4F). Consequently, we discovered that CXCl2 upregulates the neutrophil-killing program and could serve as a promising candidate for enhancing mucosal defense and managing recurrent infections.

## Discussion

We characterized mouse cervical resident macrophage subsets under various hormonal contexts and in the presence of a variety of pathogens. Here we show that P4 upregulates cervical macrophage crosstalk to promote neutrophil-killing competency. P4 presence is necessary to induce TNFA expression in MdM-pat, which licenses YsM to produce CXCl2 to activate neutrophil microbicide capacity regardless of the presence of pathogens. This mechanism promotes a wave of hostile neutrophils after ovulation, aimed at clearing pathogenic microorganisms that may infiltrate the female mucosa via semen or hide among the commensal microbiota.

Cervical macrophages, which are the dominant tissue immune cell population among vaginal and cervical mucosa (35, 36, 43), play a crucial role in coordinating cervical mucosa defense against sexually transmitted (such as chlamydia, gonorrhea, genital warts, etc.) and opportunistic diseases (such as candida), while allowing for reproduction. Macrophages were originally described as professional phagocytes; however, macrophages are not solely effector cells. They also contribute to tissue homeostasis and repair, and they play a critical role in regulating neutrophil extravasation and killing capacity. Tissue-resident macrophages continuously capture, phagocytose, and analyze surrounding material in a “silent” immunological manner (i.e., without triggering inflammation) (44, 45). When macrophages detect microbial products, they initiate the inflammation program, producing chemokines and cytokines (such as IFNS, CXCL1, CCL2, TNFA, CXCl2, IL1B) necessary for recruiting leucocytes and upregulating neutrophil-killing capacity to resolve the infection (46–49). The coordinated interplay between macrophages and neutrophils is crucial for the effective elimination of harmful agents and the restoration of tissue homeostasis after injury or infection (22, 42).

E2 levels attenuate cervical mucosa immunity during ovulation (35, 36) to allow for sperm survival, although it might predispose women to infection (12). Here we confirm that contrary to other tissues, sex hormones, rather than cervical macrophages, drive neutrophil tissue infiltration (8, 41). Moreover, during ovulation, MdM-pat were unable to trigger the neutrophil-killing program even when pathogens were present in the vaginal lumen. E2 inhibits NF-κB nuclear translocation, which modulates macrophage function by impairing the expression of proinflammatory cytokines such as TNFA (50) and chemotaxis (36, 51). Indeed, fertile women with high estrogen levels during their menstrual cycle are most susceptible to pathogenic infections (52). Nonetheless, following ovulation, P4 restores cervical mucosa immunity not only by activating neutrophil infiltration (8) but also by increasing the MdM-pat subset presence and TNFA expression; this consequently induces YsM to produce CXCl2, thereby initiating the neutrophil-killing program in a P4-dependent manner and independent of the presence of pathogens. Our data indicate that, to protect sperm during ovulation, the infiltration of MdM-pat is minimal, and they are ineffective at inducing microbicidal neutrophils, although this activity increases in the presence of pathogens. However, after ovulation, P4 triggers female mucosal immunity to promote hostile neutrophils to clear up pathogenic microorganisms that could seize semen or grow among the commensal microbiota during ovulation. Therefore, hormonal deregulations may lead to infertility due to neutrophil-mediated sperm attacks or may compromise vaginal immunity, rendering it more susceptible to infections.

In our investigation, we demonstrate that YsM produces CXCl2, which contributes to the activation of the neutrophil-killing program. While conducting CXCl2 knockdown experiments would be valuable for validation, their execution is intricate due to CXCl2’s dual role in guiding neutrophil passage through endothelial cells (53). Consequently, CXCl2 knockdown would impede neutrophil migration to the vaginal lumen, thus hindering the assessment of their microbicidal function. We employed a combined hormone model to simulate ovarian cycle phases, allowing us to investigate the effects of sex hormones more accurately. Nevertheless, the use of clodronate liposomes to deplete macrophage populations in tissues remains controversial (54). Although we observed no effect of clodronate on vaginal neutrophil migration, clodronate-treated mice exhibited reduced killing capabilities, potentially attributable to MdM-pat depletion. In addition, an understanding of how mucosal immunity failure allows early colonization of pathogenic organisms in human tissues remains limited. Human studies mainly focus on preestablished vaginal infections due to the lack of an appropriate model to assess infections in a standardized manner. Despite the differences between humans and mice in anatomy, physiology, diet, and vaginal mucosal relationship with microbes (55), which implies variances in vaginal pH levels (56), rodents continue to serve as robust and clinically relevant animal models for studying vaginal infections. The ongoing optimization of humanized mouse models, aiming to establish the functional role of the mucosa in health and disease, allows for the improvement of preclinical models for therapeutic interventions.

Vaginal pathogens have evolved to either circumvent or exploit the E2-dependent sperm protection program during ovulation or persist in the vaginal tract and infect opportunistically. Here, we offer insights into how P4 activates vaginal innate immunity in response to opportunistic pathogens that could grow during the ovulatory phase and whether P4 dysregulations could result in a suboptimal neutrophilic response, leading to insufficient mucosal defense and increased risk of unsolved infections. Therefore, we suggest that CXCl2’s role in the neutrophil antimicrobial program could be utilized as an adjuvant in the treatment of recurrent vaginal infections. However, further studies are needed to improve our understanding of how CXCl2 affects the pathophysiological functions of vaginal and cervical macrophages and neutrophils in order to apply such knowledge to the treatment of recurrent vaginal infections.

## Methods

### Sex as a biological variable.

Due to the specific focus on cervical and vaginal immune responses, only females were included in this study.

### Vaginal cytology.

Eight-week-old female BALB/c mice (H-2d) were maintained under specific pathogen–free, environment-enriched, temperature/humidity-controlled conditions and 12-hour light/dark settings, in the Animal Facility of IiSGM. To analyze ovarian cycle stages, we gently placed 10 μL of PBS into the vaginal orifice; we flushed, collected, and placed the retrieved vaginal fluid on a glass slide, which was then observed by light microscopy with a 10× objective. Proestrus and metestrus phases in mice were considered follicular phase and luteal phase, respectively (57), per hormonal profiles.

### Hormonal treatment.

To mimic female ovarian cycle hormones, mice were bilaterally ovariectomized under anesthesia. After 2 weeks of recovery, they were injected s.c. with 0.006 mg of 17β-E2 (Calbiochem) dissolved in 100 μL of sesame oil (Sigma-Aldrich) 72 hours before being challenged. Twenty-four hours before infection, mice were treated with 0.2 mg of P4 (Calbiochem) or 17β-E2 (8). For single-hormone treatments, ovariectomized mice were treated either with 0.2 mg of P4 (Calbiochem) or 17β-E2 dissolved in 100 μL of sesame oil (Sigma-Aldrich) 72 hours before being challenged (58). Hormonal treatment was sufficient to maintain hormone concentrations (10). Proestrus mice were injected s.c. with 100 μL of 25 mg Prolutex (IBSA Farmaceutici) for P4 treatment. Then, 12 hours later, mice were sacrificed, and cervical and vaginal tissues were analyzed.

### Culture of microorganisms, and vaginal killing assay.

*C. albicans* (ATCC MYA-2876) strain was grown on Sabouraud dextrose chloramphenicol agar plates (Conda) overnight at 30°C prior to the experiments (7). *T. vaginalis* (ATCC, C-1:NIH) strain was grown on TYM medium for 48 hours at 37°C in a 5% CO_2_ atmosphere before the experiments (59). *N. gonorrhoeae* (ATCC, 700825) was grown on CG agar plates at 35°C–37°C in a 5% CO_2_ atmosphere for 48 hours (60).

*N. gonorrhoeae* (2 × 10^6^ CFU), *T. vaginalis* (2 × 10^6^), and *C. albicans* SC5314 (2 × 10^6^) (58) were inoculated vaginally in PBS (20 μL) for 12-hour infections (8). Mice were sacrificed and vaginal secretions were gently collected by flushing the vagina 4 times with 50 μL of sterile PBS (7, 58). Vaginal samples were serially diluted 10-fold and live *C*. *albicans* were assayed on Yeast-Extract Dextrose Medium (YED, Conda) agar plates while live *T*. *vaginalis* were assayed through trypan blue staining and counted in a Neubauer chamber.

### Ex vivo killing assay.

Neutrophils were isolated from BM by an anti-Ly-6G kit (Miltenyi Biotec) following the manufacturer instructions. Neutrophils were pulsed for 1 hour with TNFA (Peprotech), CXCl2 (Peprotech), E2, or P4 at the concentration indicated in the appropriate figure legend before they were challenged at MOI of 1 for 2 hours at 37°C in 5% CO_2_. After coculturing, *C*. *albicans* Triton X-100 (0.1% final concentration; Sigma-Aldrich) was added to the wells, and the plates were shaken vigorously. Serial dilutions from each well were prepared in distilled water and plated (triplicate samples) on YED agar plates (61). *T*. *vaginalis* were assayed through trypan blue (Sigma-Aldrich) staining and counted in a Neubauer chamber. Sperm viability detections were performed with the live/dead sperm viability kit (Thermo Fisher Scientific) following the manufacturer instructions (12). Sperm viability detections were performed with the live/dead sperm viability kit (Thermo Fisher Scientific) following the manufacturer instructions by confocal microscopy or flow cytometry (12).

### Clodronate liposomes, inhibitors, and proteins.

To deplete MdM-pat macrophages, we administrated 0,1 mL of 5 mg/mL of clodronate-encapsulated liposomes and PBS-encapsulated liposomes (Clodronate Liposomes) i.v. 3 days before the *C*. *albicans* vaginal challenge.

We examined E2/P4-treated and E2/E2-treated mice at 12 hours and 24 hours, respectively, following the *C*. *albicans* vaginal challenge. Notably, we observed the presence of vaginal neutrophils in E2/E2 mice during this time (Figure 1C; Figure 2, C and D; and Supplemental Figure 4G). Consequently, the absence of candidacidal activity in these mice cannot be attributed to a lack of neutrophils. We assessed the effect of clodronate by analyzing the density of Mo-pat and MdM-pat. Notably, we observed a significant reduction in MdM-pat density, although the overall density of tissue-resident macrophages remained similar (Supplemental Figure 3, B–F). However, we detected the same number of vaginal neutrophils in both mock- and clodronate-treated mice in the vaginal lavage, which validates our analysis of the MdM-pat depletion effect on the neutrophils’ candidacidal function (Figure 2, C and D) (54).

P4-treated ovariectomized mice were injected i.p. with etanercept (3 mg/kg, Pfizer) every other day for 5 days preceding the *C*. *albicans* challenge. In total, 160 ng of CXCl2 (R&D Systems) in 10 μL of PBS was used to stimulate vaginal neutrophils.

### Flow cytometry.

Cellular phenotypic analysis was carried out by direct immunofluorescence. All incubations were done in the presence of 50 μg/mL mouse IgG. The same isotype control antibody was always included as a negative control, and dead cells were excluded by Fixable Viability Dye eFluor 450 (eBioscience). Vaginal single-cell suspensions were obtained through the Tumor Dissociation Kit, mouse (Miltenyi Biotec), following the manufacturer’s instructions. Flow cytometry was performed with a Gallios device (Beckman Coulter), and cells were counted using Flow-Count fluorospheres (Beckman Coulter) following the manufacturer’s instructions and FACS gating strategies for tissue macrophages and blood monocytes (Supplemental Figure 1D and Supplemental Figure 3B). For intracellular staining, we used the BD Cytofix/Cytoperm Fixation/Permeabilization kit (BD Biosciences) following the manufacturer’s instructions. Antibodies for flow cytometry include the following: CD45 (30-F11, Invitrogen), F4/80 (BM8, BioLegend), MHCII (REA813, Miltenyi Biotec), FOLRB2 (10/FR2, BioLegend), CCR2 (SA203G11, BioLegend), CCR2 (REA538, Miltenyi Biotec), TNFA (MP6-XT22, BioLegend), CXCl2 (AF-452-SP, R&D Systems), LY6G (1A8, BioLegend), CD11B (M1/70, BioLegend), LY6C (HK1.4, eBioscience), and CX3CR1 (SA011F11, BioLegend).

### Confocal microscopy.

FRT tissues were embedded in Tissue-Tek OCT (Sakura). Sections (8 μm) were fixed with acetone, blocked (50 μg/mL mouse IgG and 10% FBS), and stained. To screen the cervical cells and protein expression in vivo quantification, tissues were consistently triple stained and imaged using the glycerol ACS APO 20× NA 0.60 immersion objective on a confocal fluorescence microscope (SPE, Leica Microsystems). Acquisition settings remained consistent throughout the process for each sample and across samples, following previously described protocols (8, 10). Two to 4 pictures of the cervix were analyzed per mouse. Mean fluorescence intensities (MFI) and cell number were evaluated at multiple pictures (2–4 pictures per mouse), typically comprising stromal area and excluding the cervical epithelium. All quantifications, including line profiles, were performed using the FIJI software (NIH). Antibodies for confocal microscopy include the following: CD45 (30-F11, Invitrogen), F4/80 (BM8, BioLegend), MHCII (M5/114.15.2, BioLegend), FOLRB2 (10/FR2, BioLegend), TNFA (MP6-XT22,BioLegend), CXCl2 (AF-452-SP, R&D Systems), CCR2 (SA203G11, BioLegend), E2 receptor (ERA) (H-184, Santa Cruz Biotechnology Inc.), P4 receptor (PGR) (C-19, Santa Cruz Biotechnology Inc.), and CX3CR1 (SA011F11, BioLegend).

### Statistics.

The test used to determine the statistical significance between treatments in each experiment can be found in the figure legends; Kruskal-Wallis analysis was used. For this, we used GraphPad Prism 5 (GraphPad Software) and IBM SPSS Statistics for Windows (IBM Corp.). *P* < 0.05 was considered statistically significant.

### Study approval.

All the procedures followed and applied in this study were approved by the IiSGM Animal Care and Use Committee and the Comunidad de Madrid (PROEX-120/17, 188/18 and 198/19).

### Data availability.

Values for all data points in graphs are reported in the Supporting Data Values file.

## Author contributions

MR conceived the study. CGO, MCL, PAP, NLE, KRBC, JGV, MMP, and MR carried out the experiments. AIE, EM, and LALF helped with pathogens infection experiments, sample collection key reagents, and protocols. CGO, MCL, and MR analyzed the data. MR wrote the manuscript, and MMP, EM, MP, LALF, and FPM revised the manuscript. All the authors approved the submitted version.

## Supplementary Material

Supplemental data

Supporting data values

## Version 1 09/19/2024

In-Press Preview

## Version 2 10/22/2024

Electronic publication
