# Supplementary material for: Progesterone promotes CXCl2-dependent vaginal neutrophil killing by activating cervical resident macrophage–neutrophil crosstalk
Source: JCI Insight. 2024 Oct 22;9(20):e177899. doi: 10.1172/jci.insight.177899 (PMC11529979; doi:10.1172/jci.insight.177899)
Supplement: Supplemental data [file jciinsight-9-177899-s299.pdf]

Supp.Fig. 1

A

	F4/80	FOLR2	CCR2	MHC II	CXCR1	CX3CR1	CD11C	LY6C
Mo-Inf	+	-	+++		+	-/+		++
Mo-Pat	+	-	-/+		++	++	+	-/+
YdM	++	++	-	+		+		
MdM-inf	++	-	++	++		++		
MdM-pat	++	-	-	+++	++	+++	+	-

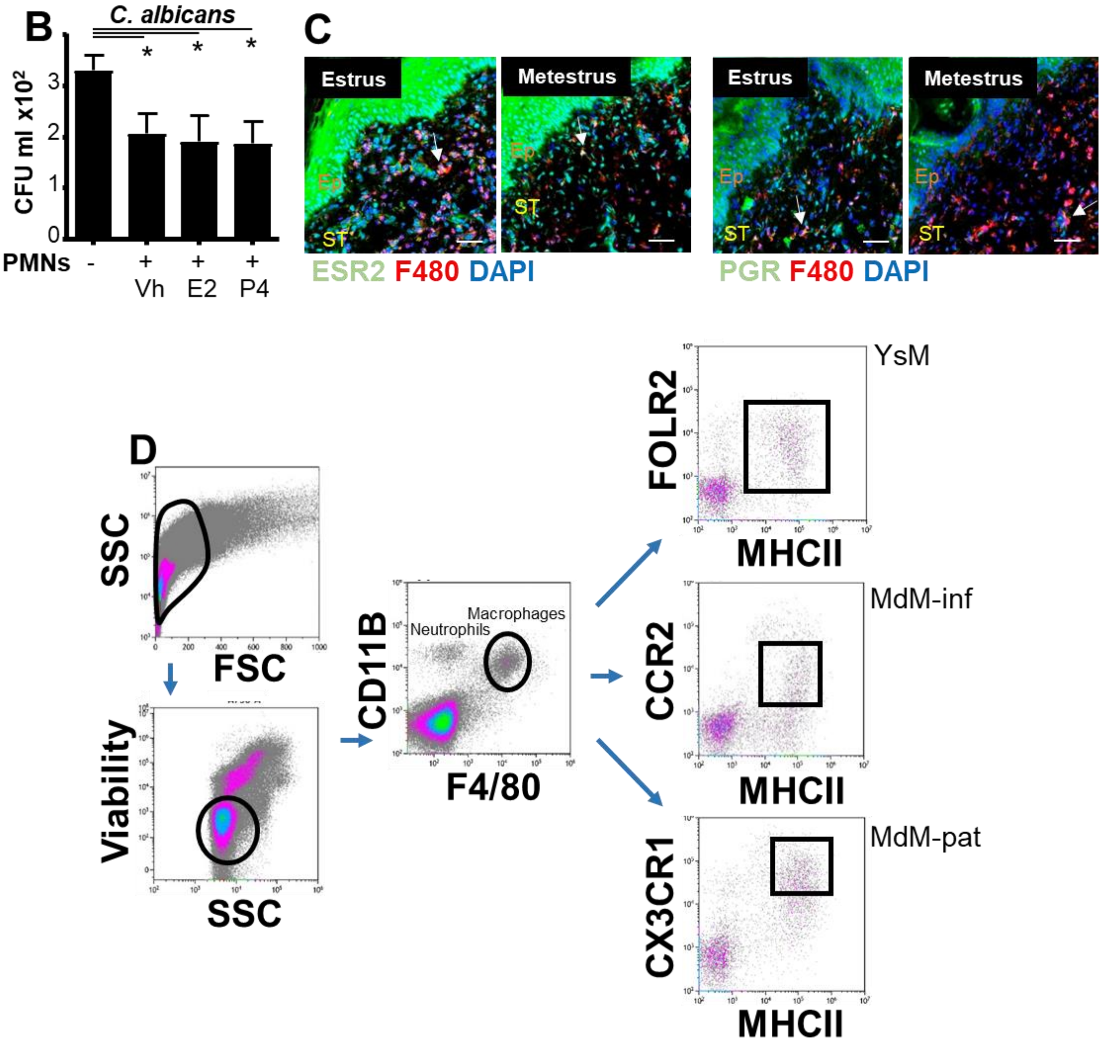

Supplemental data Figure 1. (**A**) Markers of different populations of monocytes and macrophages. Mo-inf: inflammatory monocytes, Mo-pat: patrolling monocytes, YdM: yolk sac-derived macrophages, MdM-inf: macrophages derived from inflammatory monocytes and MdM-pat: macrophages derived from patrolling monocytes. Mann-Whitney test. (**B**) Alive C. albicans co-cultured with ex vivo neutrophils treated with E2:estradiol (10^{-10}M), P4:progesterone (10^{-8}M), and Vh:vehicle. Representative experiment of 3 technical repeats expressed as bar charts ±SD . Mann-Whitney. (**C**) Photomicrograph of the ectocervix of estrus and metestrus stage mice. Estradiol (ESR2) and progesterone (PGR) receptor expression in macrophages (F4/80+). White arrow points to ESR2 or PGR expression on F4/80 cells. Mann-Whitney test. (**D**) Vaginal lumen macrophage analysis by Flow cytometry. *$p<0.05$. Scale bar, 50 µm. Ep:epithelium; St:Stroma; CFU:colony-forming unit

Supp.Fig. 2

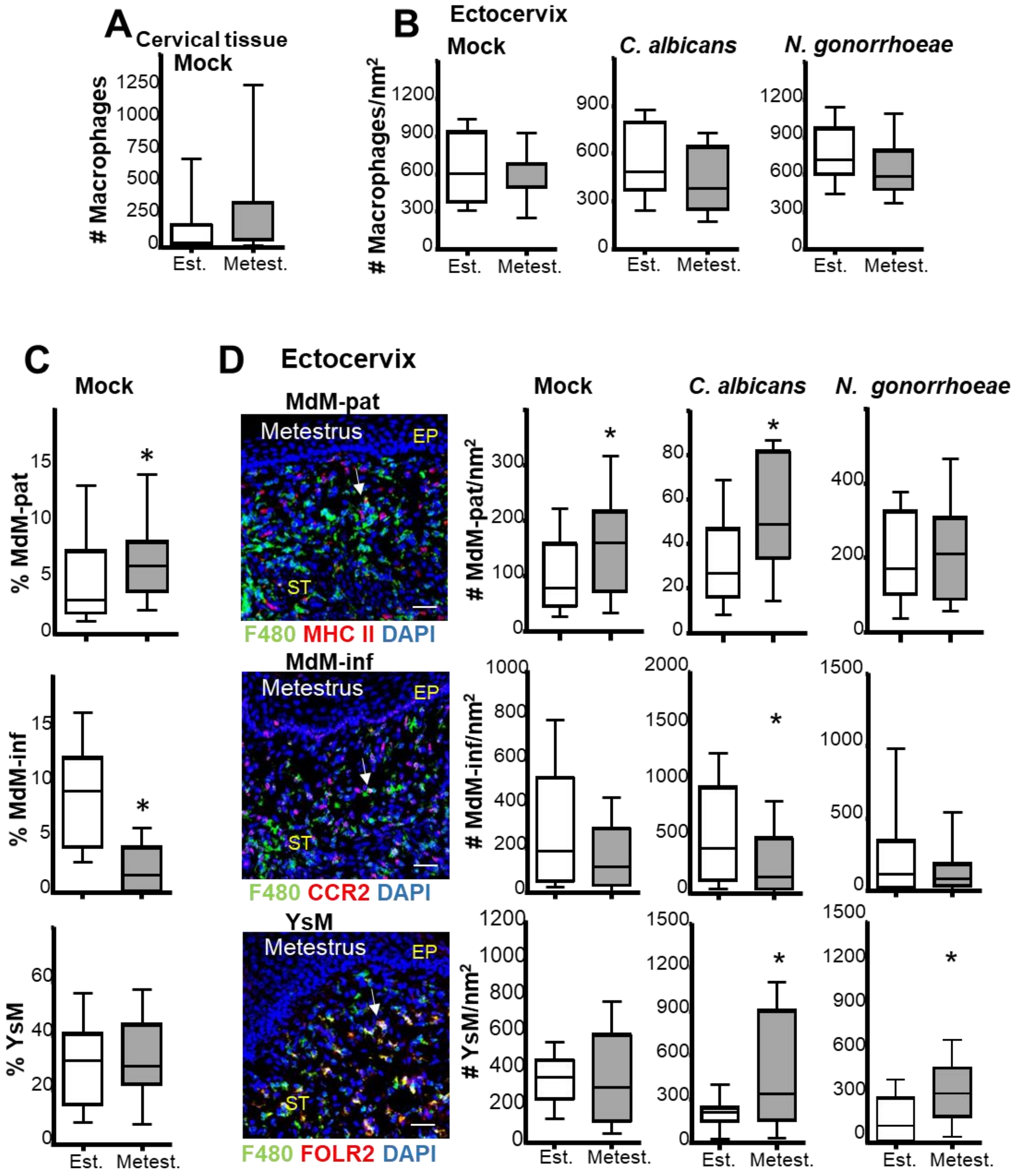

Supplemental data Figure 2. Adult female mice selected by vaginal smear assayed for: (**A**) macrophages (CD45-F4/80) in the vaginal tissue by flow cytometry and (**B**) cervix by confocal microscopy in mock, *N. gonorrhoeae* and *C. albicans* infected mice. Mann-Whitney test. Macrophage subset density in the vaginal tissue by flow cytometry (**C**) and cervix by confocal microscopy (**D**) in mice selected by vaginal smear. MdM-pat: patrolling monocytes derived macrophages (F4/80-MHCII-CX3CR1), MdM-inf: inflammatory monocytes derived macrophages (F4/80-CCR2) and YsM: Yolk sac derived macrophages (F4/80-FOLR2). Mann-Whitney test. Confocal data were calculated in 2 to 4 different sections of each sample. Data were calculated in at least 3 experiments (n=4-12 mice per group) and expressed as box and whiskers 10-90 percentile. *p<0.05, Mann-Whitney. Scale bar, 50 µm. Ep:epithelium; St:Stroma; CFU:colony-forming unit; Est:Estrous; Metest:Metestrus.

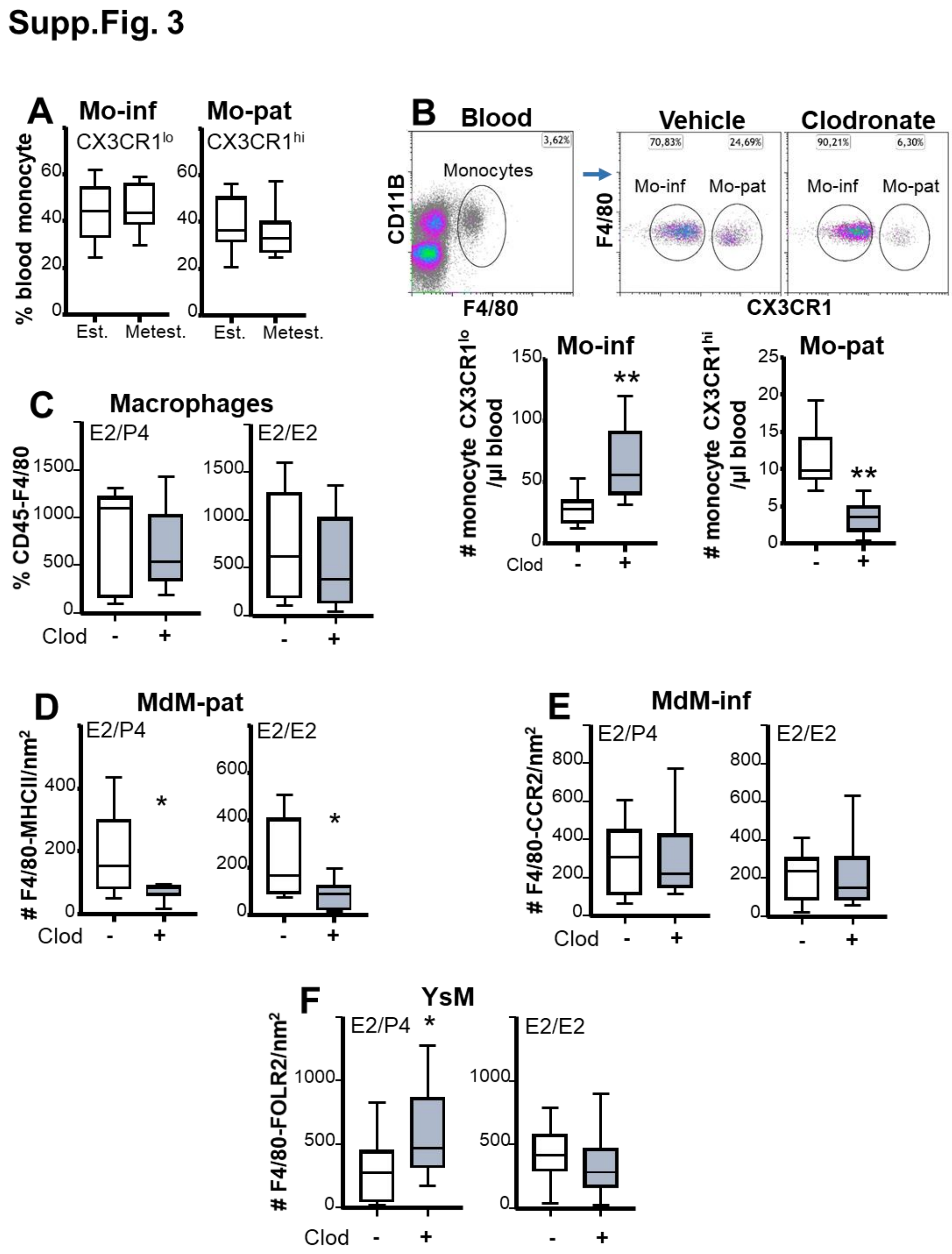

Supplemental data Figure 3. (**A**) Number of Mo-inf: inflammatory monocytes (F4/80-CX3CR1^{lo}) and Mo-pat: patrolling monocytes (F4/80-CX3CR1^{hi}). Ovariectomized mice treated with clodronate liposomes (clod), and estradiol or progesterone to mimic the ovarian cycle challenged in the vagina with *Candida albicans*. (**B**) Number of Mo-inf and Mo-pat in the blood by flow cytometry. Number of (**C**) macrophages (CD45-F4/80), (**D**) MdM-pat (F4/80-MHCII-CX3CR1), (**E**) MdM-inf (F4/80-CCR2) and (**F**) YsM (F4/80-FOLR2) in mock and clodronate liposomes (clod) treated mice. Confocal data were calculated in 2 to 4 different sections of each sample. Data were calculated in 2 or 3 experiments (n=5-12 mice per group) and expressed as box and whiskers 10-90 percentile. *$p<0.05$ and **$p<0.01$, Mann-Whitney. E2:estradiol; P4:progesterone; CFU:colony-forming unit; Est:Estrous; Metest:Metestrus; YsM: Yolk sac derived macrophages; MdM-pat: patrolling monocytes derived macrophages; MdM-inf: inflammatory monocites derived macrophages.

Supp.Fig. 4

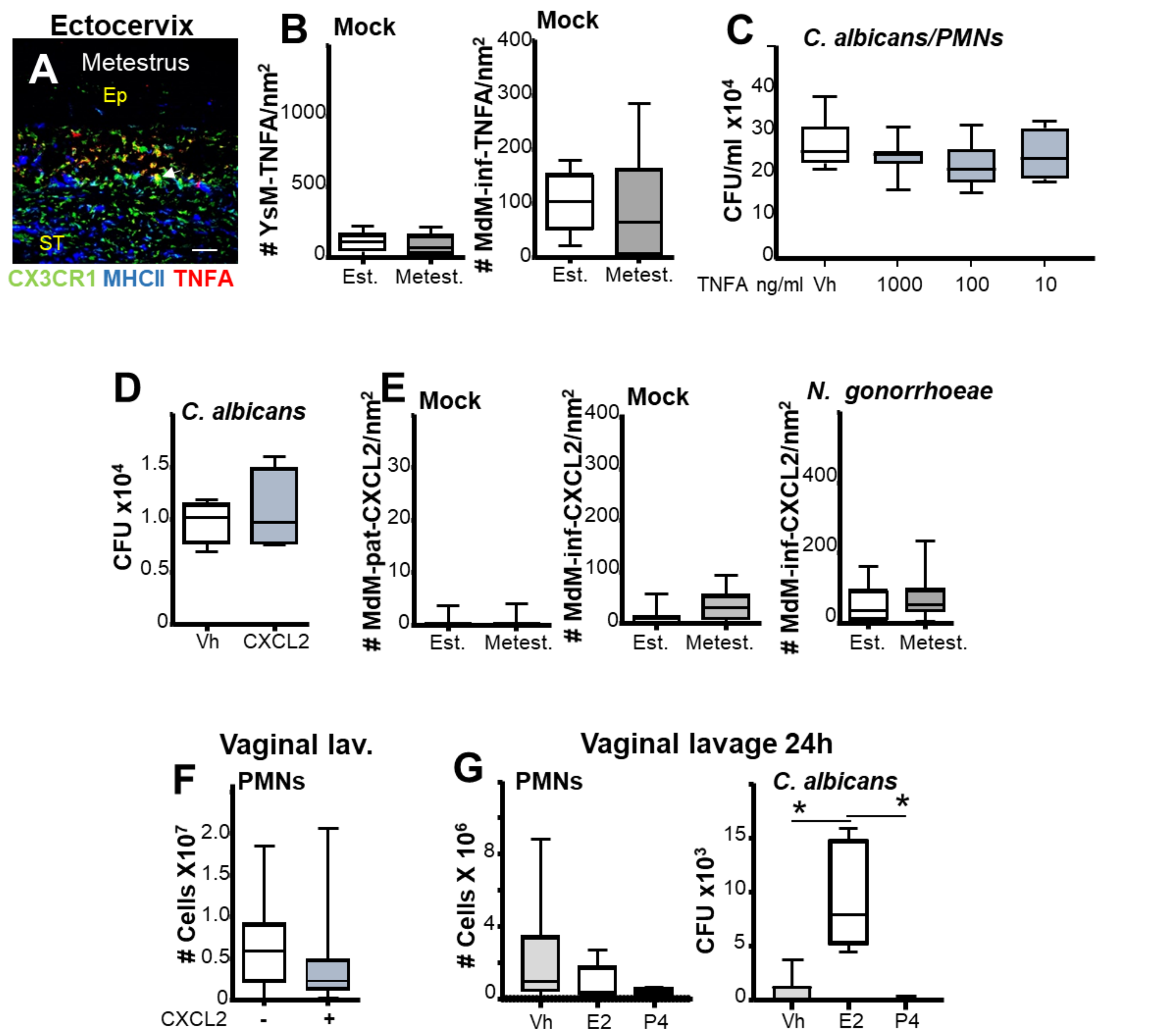

Supplemental data Figure 4. (**A**) Photomicrograph of the ectocervix of metestrus mice selected by vaginal smear. White arrow points to TNFA expression on MdM-pat (CX3CR1-MHCII). (**B**) Adult female mice selected by vaginal smear assayed for TNFA expression in cervical YsM (F4/80-FOLR2) and MdM-inf (F4/80-CCR2) by confocal microscopy. (**C**) Number of alive *C. albicans* after 2h of co-culture with ex vivo neutrophils treated with TNFA. (**D**) Number of alive *C. albicans* treated with CXCL2. (**E**) CXCL2 expression in cervical MdM-pat (F4/80-MHCII) and MdM-inf (F4/80-CCR2) by confocal microscopy. (**F**) Number of neutrophils analyzed by flow cytometry from ovariectomized mice challenged in the vagina with *C. albicans* and treated with CXCL2 (160ng) 2h after the infection (from Figure 5D). (**G**) E2:estradiol- P4:progesterone- or Vh:vehicle-treated mice challenged in the vagina with *C. albicans* for 24h. Confocal data were calculated in 2 to 4 different sections of each sample. Data were calculated in 2 or 3 experiments (n=5-9 mice per group) and expressed as box and whiskers 10-90 percentile. *p<0.05, Mann-Whitney. CFU:colony-forming unit; Est:Estrous; Metest:Metestrus; YsM: Yolk sac derived macrophages, MdM-inf: inflammatory monocytes derived macrophages and MdM-pat: patrolling monocytes derived macrophages.
